# MicroRNA-302b Enhances the Sensitivity of Hepatocellular Carcinoma Cell Lines to 5-FU via Targeting *Mcl-1* and *DPYD*

**DOI:** 10.3390/ijms161023668

**Published:** 2015-10-06

**Authors:** Donghui Cai, Kang He, Su’e Chang, Dongdong Tong, Chen Huang

**Affiliations:** Department of Genetics and Molecular Biology, Xi’an Jiaotong University College of Medicine, Xi’an 710061, China; E-Mails: yiqiu9999@stu.xjtu.edu.cn (D.C.); hek.kq.04@stu.xjtu.edu.cn (K.H.); sue4111015007@stu.xjtu.edu.cn (S.C.); tongdong@stu.xjtu.edu.cn (D.T.)

**Keywords:** miR-302b, 5-Fluorouracil (5-FU), sensitivity, hepatocellular carcinoma cell lines

## Abstract

MiR-302b is a member of miR-302-367 cluster. The miR-302-367 cluster played important roles in maintaining pluripotency in human embryonic stem cells (hESCs) and has been proved to be capable of suppressing cell growth in several types of cancer cell lines including Hepatocellular Carcinoma (HCC) Cell lines. However, the role that miR-302b plays in the 5-Fluorouracil (5-FU) sensitivity of HCC has not been known. This study showed that miR-302b could enhance the sensitivity to 5-FU in HCC cell lines and verified its two putative targeted genes responsible for its 5-FU sensitivity.

## 1. Introduction

HCC is one of the most common types of cancers, and ranks among the cancers of the highest mortality rates in China [1]. Among more than one hundred types of drugs used to treat HCC, 5-FU is for now the most optimal one, and it is widely used. However, 5-FU monotherapy is not very effective in the treatment of advanced HCC due to the rapid development of 5-FU resistance. Therefore, many people are committed to seeking the genes that can influence the sensitivity of 5-FU in cancer cells, or the cell apoptosis induced by 5-FU. *Mcl-1,* a member of the Bcl-2 family, is highly expressed in various malignant tumors, and plays an important role in inhibiting apoptosis [2,3,4]. An unstable nature was bestowed upon *Mcl-1* by its short half-life , which provides the mechanism for cells to switch into growth or apoptosis state in reaction to various forms of stresses [5,6]. Some reports have shown that the RNA interference of *Mcl-1* could sensitize cancer cells to chemotherapy [7,8]. In addition to *Mcl-1*, other genes involved in 5-FU metabolism could also influence the sensitivity of 5-FU in cells. Since about 80% of 5-FU is catabolized by dihydropyrimidine dehydrogenase (*DPYD*), the initial and rate-limiting enzyme of pyrimidine catabolism, only a small part of the 5-FU can inhibit thymidylate synthase (TS) so as to cause cancer cell death via thymineless death [9,10]. Some types of cancers exhibited high expression levels of *DPYD* [11], and some reports demonstrated that the up-regulation of *DPYD* in HCC cell lines could decrease the sensitivity to 5-FU [12], and *vice versa* [13]. Hence, to some extent, the level of *DPYD* could influence the sensitivity of 5-FU, and it may become an important factor to mould5-FU resistance.

In addition to the two genes mentioned above, some studies have shown that microRNAs got involved in 5-FU sensitivity. MicroRNAs (miRNAs) are a class of endogenous 20 to 25-nucleotide non-coding RNAs that negatively regulate the expression of their complementary messenger RNAs (mRNAs) in eukaryotes, exerting influence on various biological processes like development, differentiation, apoptosis, and carcinogenesis [14,15,16]. Many of the miRNAs are connected with carcinogenesis, and some of them have the potential of being the important molecules influencing the cancer therapy [17]. Recently, some miRNAs were found to influence the 5-FU sensitivity via targeting 5-FU metabolic enzymes. For example, miR-433 binding to the 3′ untranslated region (3′ UTR) of TYMS mRNA influence the 5-FU sensitivity in HeLa cells [18].

The miR-302b lies in the miR-302-367 cluster, where else includes miR-302c, miR-302a, miR-302d and miR-367 [19]. The miR-302-367 cluster was found to play an important part in maintaining pluripotency in hESCs [20,21,22], reprogramming somatic cells into induced pluripotent stem cells (iPSCs) [23,24], inhibiting the tumorigenecity of human pluripotent stem cells [25], and suppressing cancer cell proliferation [26,27].

In our study, we observed the miR-302b’s function of suppressing proliferation in human hepatoma cell lines and found that ectopic overexpression of miR-302b could enhance the sensitivity of HCC to 5-FU by negatively regulating *DPYD* and anti-apoptosis protein *Mcl-1*, which suggests that miR-302b might serve as a therapeutic reagent for HCC.

## 2. Results and Discussion

### 2.1. MiR-302b Represses Cell Proliferation and Cell Cycle Progression in Liver Cancer Cell Lines

**Figure 1:**
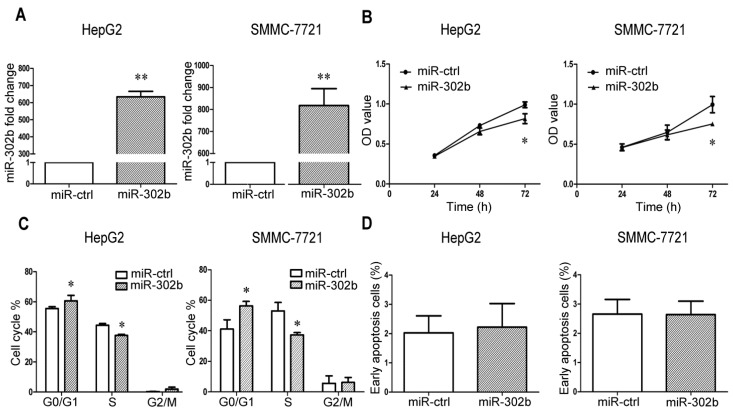
MiR-302b represses cell proliferation, cell cycle progression in liver cancer cell lines. (**A**) qRT-PCR showed that a significant increase after infection of miR-302b vector into HepG2/SMMC-7721, miR-ctrl as negative control; (**B**) MiR-302b suppressed the cell proliferation of HepG2/SMMC-7721, which was determined by MTT assay; (**C**) Overexpression of miR-302b in HepG2/SMMC-7721 blocked the cell cycle transition from G0/G1 to S phase; (**D**) Overexpression of miR-302b in HepG2/SMMC-7721 did not show obvious difference in apoptosis compared with the control cells. Error bars represent the S.D; * *p* < 0.05; ** *p* < 0.01, *vs.* the corresponding controls.

**Figure 2:**
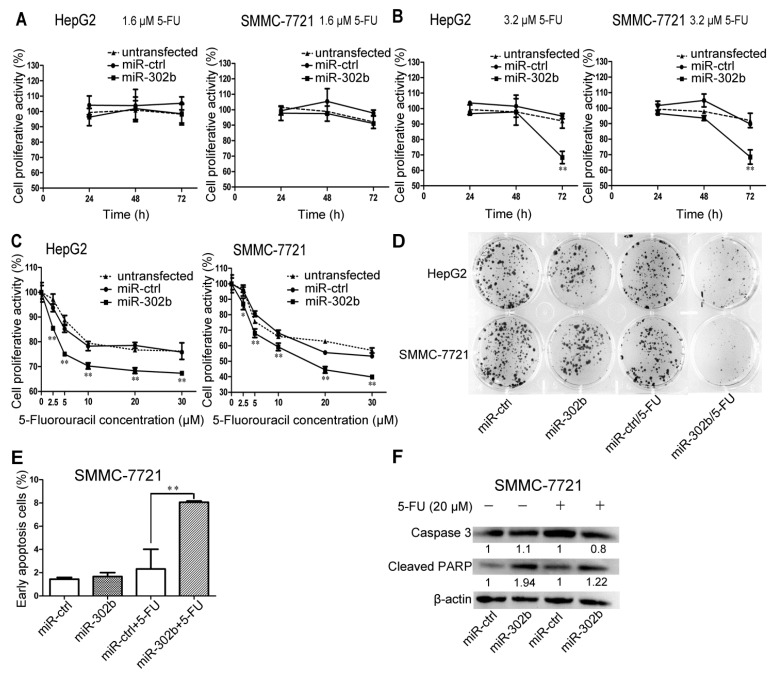
MiR-302b enhances the sensitivity to 5-FU in HepG2 and SMMC-7721 cells in a time- and dose- dependent manner. (**A**,**B**) 5-FU chemosensitivity was measured by MTT assay in miR-302b, miR-ctrl transfected or untransfected HepG2/SMMC-7721 cells treated with 1.6 μM 5-FU (**A**) or 3.2 μM 5-FU (**B**) for 24, 48 and 72 h. MiR-ctrl as the control; (**C**) Effect of miR-302b on chemosensitivity to 5-FU over 3.2 μM was measured at 72 h by MTT assay in HepG2/SMMC-7721 cells. MiR-ctrl as the control; (**D**) HepG2/SMMC-7721 cells were treated with miR-ctrl, miR-302b, with or without a combination of 3.2 μM 5-FU in the colony formation assays; (**E**) PI/Annexin V staining FACS analyses measured apoptosis induced or not by 5-FU in SMMC-7721 cells transfected with miR-302b or miR-ctrl; (**F**) Western blot of caspase3 and cleaved PARP detected apoptosis induced or not by 5-FU in SMMC-7721 cells transfected with miR-302b and miR-ctrl. Error bars represent the S.D.; * *p* < 0.05; ** *p* < 0.01, *vs.* the corresponding controls.

In order to get a general idea of the function of miR-302b in liver cancer cell lines, miR-302b expression plasmid and the control plasmid were respectively transfected into the HepG2/SMMC-7721 cells. The result of real-time PCR showed that a huge increase of miR-302b expression level occurred 48 h later in HepG2/SMMC-7721 cells infected with pre-miR-302b plasmid (Figure 1A), partly due to comparing with the very low expression level of miR-302b in HepG2/SMMC-7721 cells transfected with control plasmid. This very low expression level of miR-302b in liver cancer cell lines also caused little change of miR-302b expression after transfection of miR-302b inhibitor into SMMC-7721 cells. After overexpression of miR-302b in HepG2/SMMC-7721, we observed an obvious suppression of cell proliferation by the MTT (Figure 1B) and colony formation assays (Figure 2D). To determine whether miR-302b-induced cell growth suppression was associated with cell-cycle block, we performed the FACS to measure the cell-cycle phase distribution. Overexpression of miR-302b in HepG2/SMMC-7721 caused an increase of cells in G0/G1-phase population and a decrease of cells in S-phase population, which indicated that miR-302b blocked the transition from G0/G1 to S phase in HCC cells (Figure 1C). The above observations were consistent with the results of previous studies [26,27]. We further explored the role played by miR-302b in cell apoptosis, and found that the miR-302b transfected cells showed no significant difference in apoptosis (Figure 1D).

### 2.2. Overexpression of miR-302b Enhances the Sensitivity to 5-FU in HepG2 and SMMC-7721 Cells

In order to investigate the 5-FU sensitivity of the HCC cell lines influenced by miR-302b, the miR-302b, miR-ctrl transfected or untransfected HepG2/SMMC-7721 cells were treated with 5-FU at low concentrations of 0, 0.2, 0.4, 0.8, 1.6, 3.2 μM. We performed MTT assays to evaluate the cell proliferation at 24, 48, and 72 h. The results showed that distinct decrease of the cell proliferation of miR-302b transfected HepG2/SMMC-7721 cells, compared with the ones transfected with miR-ctrl, which happened after treatment with 5-FU at the concentration of 3.2 μM and treatment for 72 h (Figure 2B), but proliferation was not affected at 5-FU concentrations below 1.6 μM (Figure 2A). Meanwhile, there was no difference observed between the untransfected and miR-ctrl transfected group (Figure 2A). These results suggested that overexpression of miR-302b in HepG2/SMMC-7721 cells could enhance the cells’ sensitivity to 5-FU when the concentration of 5-FU at least over 1.6 μM. Based on this result, the 5-FU concentration of 3.2 μM was adopted into the colony formation assay, which further verified miR-302b’s effectiveness on enhancement of sensitivity to 5-FU in HepG2/SMMC-7721 cells (Figure 2D).

To explore the effect of miR-302b on 5-FU sensitivity in HCC treated with 5-FU at concentration over 3.2 μM, the concentrations of 2.5, 5, 10, 20, 30 μM were set up to perform the MTT assays. As shown in Figure 2C, compared to the miR-ctrl, miR-302b significantly decreased proliferation of HCC cells treated with 5-FU, although the untransfected group did not show difference (Figure 2C).

### 2.3. MiR-302b Targets Mcl-1 and DPYD

**Figure 3:**
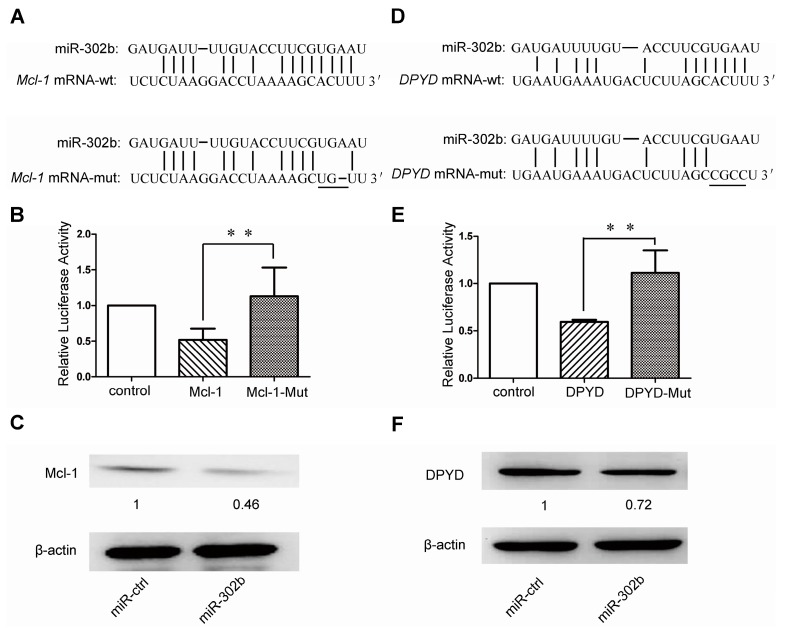
MiR-302b directly targets *Mcl-1* by binding to the 3′ UTR and *DPYD* coding region. (**A**,**D**) schematic representation of miR-302b seed sequence within the 3′ UTR of *Mcl-1* (**A**) and coding region of *DPYD* (**D**). Mutations in the seed region of miR-302b were respectively generated in the 3′ UTR of *Mcl-1* and in the CDS of *DPYD*, as indicated by underline. The target genes sequences and their mutated ones were respectively cloned into the *pmirGLO* Dual-Luciferase miRNA Target Expression vector; (**B**,**E**) Analysis of luciferase activity; (**C**,**F**) Western blot measured the expression level of Mcl-1 (**C**) and DPYD (**F**) 48 h post transfection of miR-302b. Error bars represent the S.D; * *p* < 0.05; ** *p* < 0.01, *vs.* the corresponding controls.

To search for the miR-302b target genes involved in 5-FU sensitivity, we applied the programs RegRNA2.0 [28] and/or TargetScan [29] to get *Mcl-1* and *DPYD* genes, both of which were shown to harbor good binding sites of hsa-miR-302b-3p respectively in the 3′ UTR of *Mcl-1* gene at 2225–2231 nt and the coding domain sequence (CDS) of *DPYD* gene at 925–949 nt (Figure 3A,D). To verify the directly repressive effect of miR-302b on *Mcl-1* and *DPYD* genes, these two gene sequences corresponding to miR-302b-binding sites were inserted downstream of the luciferase reporter gene. We also mutated these two miR-302b-binding sites and cloned them into the luciferase reporter plasmid, respectively. Later, we performed the luciferase reporter assays and observed a significant decrease of luciferase activity in the presence of miR-302b compared with the miR-ctrl plasmid. In addition, we also found that the reporters carrying mutant *Mcl-1* gene or mutant *DPYD* gene were not responsive to the miR-302b (Figure 3B,E). The western blots showed that ectopic overexpression of miR-302b in HepG2 cells can down-regulate the Mcl-1 and DPYD protein levels (Figure 3C,F), but mRNA levels of these two genes did not change (data not shown), which suggested that the miR-302b suppress *Mcl-1* and *DPYD* genes expression at translational level, but not transcriptional level.

### 2.4. RNA Interference-Mediated Silencing of Mcl-1 or DPYD Enhances the Sensitivity to 5-FU in HepG2 and SMMC-7721 Cells

**Figure 4:**
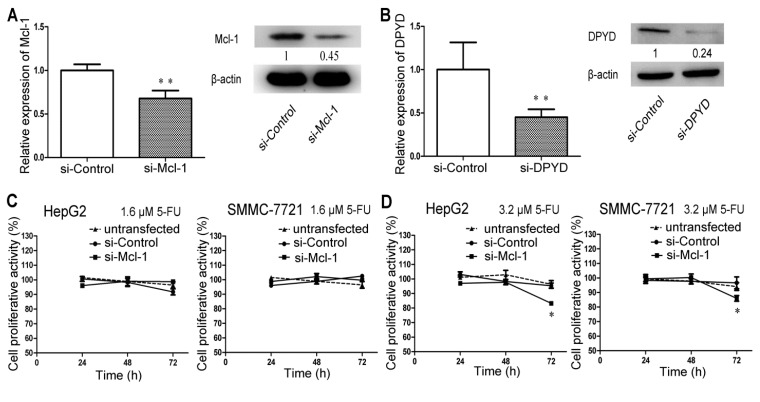

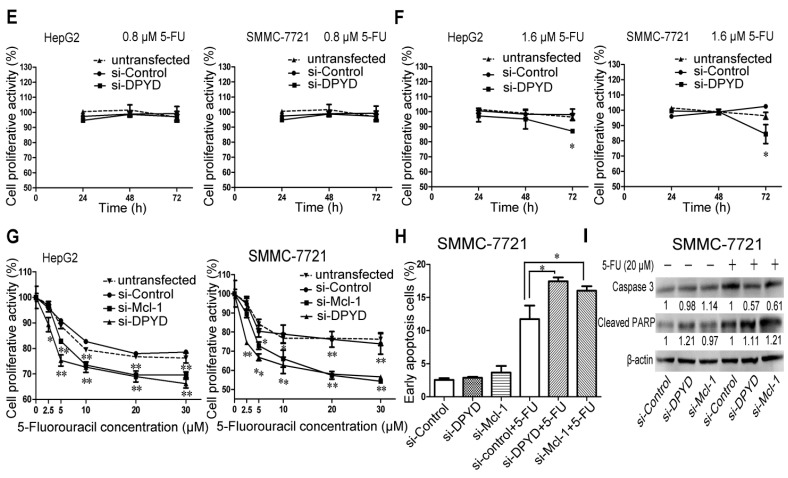
Down-regulation of *Mcl-1* or *DPYD* enhances the sensitivity to 5-FU in HepG2 and SMMC-7721 cells in a time- and dose- dependent manner. (**A**,**B**) qRT-PCR and Western blot analyses the expression level of *Mcl-1* gene (**A**) and *DPYD* gene (**B**) after transfection of si-*Mcl-1* and si-*DPYD*, respectively into SMMC-7721 cells; (**C**,**D**) 5-FU chemosensitivity was measured by MTT assay in si-Control, si-*Mcl-1* transfected or untransfected HepG2/SMMC-7721 cells treated with 1.6 μM 5-FU (**C**) or 3.2 μM 5-FU (**D**) for 24, 48 and 72 h. Si-control as the control; (**E**,**F**) Same MTT assays were performed in si-*DPYD*, si-Control transfected or untransfected HepG2/SMMC-7721 cells treated with 0.8 μM 5-FU (**E**) or 1.6 μM 5-FU (**F**). Si-control as the control; (**G**) Effect of si-*Mcl-1* and si-*DPYD* on Chemosensitivity to 5-FU over 3.2 μM was measured at 72 h by MTT assay in HepG2/SMMC-7721 cells; (**H**) PI/Annexin V staining FACS analyses measured apoptosis induced or not by 5-FU in SMMC-7721 cells transfected with si-Control, si-*Mcl-1* or si-*DPYD*; (**I**) Western blot of caspase3 and cleaved PARP detected apoptosis induced or not by 5-FU in SMMC-7721 cells transfected with si-Control, si-*Mcl-1* or si-*DPYD*. Error bars represent the S.D.; * *p* < 0.05; ** *p* < 0.01, *vs.* the corresponding controls.

Next, we also carried out the same MTT assays as performed for miR-302b to evaluate the change of sensitivity to 5-FU on HepG2/SMMC-7721 cells after transfected with *Mcl-1* siRNA or *DPYD* siRNA, which are two putative target genes of miR-302b. The siRNA control transfected HepG2/SMMC-7721 cells were set as the control group and there was no difference between the siRNA control transfected cells and the untransfected cells (Figure 4C–G). It was also observed in cells transfected with *Mcl-1* siRNA that decrease in cell proliferation was noticeable at 3.2 μM 5-FU (Figure 4D) but not 1.6 μM (Figure 4C), which was comparable with what caused by *DPYD* siRNA at 1.6 μM (Figure 4F) but not 0.8 μM (Figure 4E). Meanwhile, the 5-FU effect on cell growth inhibition could be observed after 48 h of treatment with 5-FU, regardless of *Mcl-1* or *DPYD* (Figure 4D,F). The above results maybe suggested that *DPYD* gene could play a somehow more important role in 5-FU resistance and, together with *Mcl-1* gene, partly clarified the mechanism of miR-302b enhancing the sensitivity of HepG2/SMMC-7721 cells to 5-FU. *Mcl-1* siRNA or *DPYD* siRNA transfection also significantly reduced the cell proliferation of HepG2/SMMC-7721 cells treated with 5-FU over 3.2 μM (Figure 4G), which is consistent with the results of miR-302b transfection.

### 2.5. Transfection of miR-302b Sensitizes the HCC Cell Lines to Apoptosis Induced by 5-FU

The results from apoptosis assay showed that only overexpression of miR-302b in SMMC-7721 cells hardly increase cells’ apoptosis rate (Figure 2E), but enhanced expression of miR-302b in SMMC-7721 cells led to an obvious increase in apoptosis after 5-FU (20 μM) treatment for 72 h (Figure 2E). Similar increases in apoptosis were also observed in SMMC-7721 cells after transfection of *Mcl-1* siRNA or *DPYD* siRNA in combination of 5-FU (20 μM) treatment for 72 h (Figure 4H). To further verify the results above, we detected apoptotic markers in SMMC-7721 cells by western blot after transfection and treatment with 5-FU (20 μM, for 72 h). Western blot analysis showed that transfection of miR-302b (Figure 2F) or *Mcl-1*-siRNA or *DPYD*-siRNA (Figure 4I) down-regulated the expression of caspase-3 and up-regulated the expression of cleaved PARP. These results indicated that overexpression of miR-302b enhanced the sensitivity of HCC cells to 5-FU-induced apoptosis by down-regulation of *Mcl-1* and *DPYD*.

Recently, some people began to divert attention from miR-302b’s function of maintaining pluripotency [21,22] to suppressing oncogenesis [25,26,27]. Meanwhile, Reports showed that incubation with miR-302-367 cluster for 3 weeks could reprogram HCC cells into iPSC-like-spheres by targeting AOF2. This transition from HCC into iPSC-like-spheres could sensitize HCC cells to 5-FU, which was consistent with what happened in Oct4,Sox2, Klf4, and c-Myc (OKSM)-mediated induced pluripotent stem cell (iPSC) (inserting c-Myc and random insertions of exogenous sequences into the genome) [30]. In our studies, we observed miR-302b’s effects of suppressing the proliferation of liver cancer cell lines by blocking cell cycle at G0/G1 phase (Figure 1), which was in consistence with previous reports [26,27]. Further studies indicated that the sensitivity to 5-FU could be enhanced in miR-302b-overexpressing HepG2/SMMC-7721 cells, but not in miR-ctrl-overexpressing ones (Figure 2A,B), after treatment with low concentration of 5-FU at about 3.2 μM for 72 h. It was further validated by the colony formation assay (Figure 2D). Bio-information analysis showed that 5-FU resistance-associated genes *Mcl-1* and *DPYD* both have the miR-302b-binding sites in their mRNAs. Luciferase reporter assay confirmed that miR-302b directly targeted the 3′ UTR of the *Mcl-1* (Figure 3B). However, the miR-302b binding site also proved by luciferase reporter assay lies in the coding region of *DPYD* (Figure 3E), which once more supported the opinion that the coding region and 5′ UTR, like 3′ UTR, can interact with miRNAs to produce gene silencing [31,32,33]. To further investigate the apoptosis induced by 5-FU, we performed MTT assays with the 5-FU concentration over 3.2 μM and chose the concentration of 20 μM to induce apoptosis. The results showed that overexpression of miR-302b sensitizes the HCC cell lines to apoptosis induced by 5-FU (Figure 2E). We also performed the experiments of 5-FU sensitivity and apoptosis assays on liver cancer cell lines transfected with *Mcl-1* or *DPYD* siRNA, in parallel with miR-302b tests, and got the analogous results. It suggested that miR-302b might enhance 5-FU sensitivity via its two target genes: *Mcl-1* and *DPYD* (Figure 4H).

## 3. Experimental Section

### 3.1. Cell Cultures and Chemicals

Human liver cancer cell line SMMC-7721 and HepG2 cells were cultured in 1640 medium (1640; PAA Laboratories GmbH, Pasching, Austria) with 10% fetal bovine serum (FBS) (PAA Laboratories GmbH). All cell lines were incubated at 37 °C in 5% CO2. 5-FU was bought from Sigma-Aldrich Co. LLC (Sigma F6627, New York, NY, USA). 5-FU was dissolved in PBS (Phosphate Buffered Saline), then diluted in 1640 medium at appropriate concentration. Plasmids and small interfering RNAs (siRNAs) were transfected into cells using Lipofectamine 2000 (Invitrogen, Carlsbad, CA, USA). The mount of DNA or RNA transfected and the transfection process were carried out according to the manufacturer’s instructions.

### 3.2. Plasmid Constructions

The Pre-miR-302b was chemically synthesized and cloned into pcDNA™6.2-GW/EmGFP-miR Vector harboring EGFP gene and pcDNA™6.2-GW/miR Vector without EGFP gene between the EcoRI and HindIII sites. The pcDNA™6.2-GW/EmGFP-302b plasmid was transfected into cells to estimate the transfection efficiency under the fluorescence microscopy, and the vector without EGFP was for the experiments of drug sensitivity. After bioinformatics analysis, we chemically synthesized the sequences of *Mcl-1* and *DPYD* genes’ counterparts of miR-302b-binding sites and these two genes’ mutant DNA fragments. We then cloned these fragments into *36* Dual-Luciferase miRNA Target Expression vector (Promega, Madison, WI, USA) by the restriction endonucleases XhoI and salI. All of the recombinant vectors were confirmed by sequencing. The oligonucleotides mentioned are shown in Table 1.

### 3.3. MiRA/siRNA Transfection

The miR-302b expressing plasmid, *Mcl-1* siRNA and *DPYD* siRNA were respectively transiently transfected into the HepG2/SMMC-7721 cells using Lipofectamine 2000 (Invitrogen, Carlsbad, CA, USA), 24 h after seeding. The transfection process was performed according to the manufacturer’s protocol. As negative controls, their respective control plasmid or control siRNA were also transfected into cells at the same time. The sequences of pre-miR-302b were synthesized from the Sangon Biotech (Shanghai, China). The sequences of *Mcl-1* siRNA and *DPYD* siRNA were, respectively, obtained from Adams *et al*. and Someya *et al*. [34,35] and, together with the scrambled siRNA as control, were purchased from GenePharma (Shanghai, China). The cited *Mcl-1* siRNA can not only match the full length *Mcl-1*, namely *Mcl-1L*, but also the shortened isoform of *Mcl-1*, namely *Mcl-1S*, which can bind full length *Mcl-1* and inhibit its function of anti-apoptosis [36]. However, endogenous *Mcl-1S* is expressed at low levels relative to full-length *Mcl-1L* in HCC cell lines. The expression ratios of *Mcl-1L/Mcl-1S* in HepG2 and SMMC-7721 cells are, respectively, about 1:0.036 and 1:0.023, when we usedthe *Mcl-1L*-specific primers and *Mcl-1S*-specific primers (as shown in Table 1) to detect the expression level of *Mcl-1L* and *Mcl-1S*, respectively (Figure S1A) . After transfection of *Mcl-1* siRNA into HepG2 and SMMC-7721 cells, we found that expression level of *Mcl-1L* has, respectively, decreased about 35% and 40% (Figure S1B), but *Mcl-1S* only 7% and 3% (Figure S1C). So, the inhibiting effect of this *Mcl-1* siRNA is influenced little by the *Mcl-1S*.

### 3.4. qRT-PCR Assay

Total RNA was isolated from cells using Trizol reagent (Invitrogen, USA) following the manufacturer’s instructions. First strand cDNA synthesis was performed using a PrimeScript™ RT reagent Kit (TaKaRa, Dalian, China). The cDNA was amplified using an SYBR Premix Ex Taq™ II (TaKaRa, Dalian, China). PCR amplification was performed with the IQ5 Optical System real-time PCR machine. β-actin and U6 were used for normalization of mRNA and miRNA respectively. Relative quantification of mRNA expression levels was determined using the relative standard curve method following the manufacturer’s instructions (Bio-Rad, Hercules, CA, USA). The relative expression of genes to internal control (β-actin or U6) was calculated using the 2^−(ΔΔ*C*t)^ method. The primers used are listed in Table 1.

**Table 1:** Oligonucleotides used in this study.

Name	Sequence 5′–3′
Pre-miR-302b-F	AATTCGCTCCCTTCAACTTTAACATGGAAGTGCTTTCTGTGACTTTAAAAGTAAGTGCTTCCATGTTTTAGTAGGAGTA
Pre-miR-302b-R	AGCTTACTCCTACTAAAACATGGAAGCACTTACTTTTAAAGTCACAGAAAGCACTTCCATGTTAAAGTTGAAGGGAGCG
Mcl-1-UTR-F	TCGAGGTATCTCTAAGGACCTAAAAGCACTTTATGG
Mcl-1-UTR-R	TCGACCATAAAGTGCTTTTAGGTCCTTAGAGATACC
Mcl-1-UTR-MF	TCGAGGTATCTCTAAGGACCTAAAAGCTGTTATGG
Mcl-1-UTR-MR	TCGACCATAACAGCTTTTAGGTCCTTAGAGATACC
DPYD-CDS-F	TCGAGTGAATGAAATGACTCTTAGCACTTTG
DPYD-CDS-R	TCGACAAAGTGCTAAGAGTCATTTCATTCAC
DPYD-CDS-MF	TCGAGTGAATGAAATGACTCTTAGCCGCCTG
DPYD-CDS-MR	TCGACAGGCGGCTAAGAGTCATTTCATTCAC
miR-302b-RT	GTCGTATCCAGTGCGTGTCGTGGAGTCGGCAATTGCACTGGATACGACCTACTA
miR-302b-F	TGCTTAAGTGCTTCCATGTT
miR-302b-R	ATCCAGTGCGTGTCGTG
β-actin-F	CCAACCGCGAGAAGATGA
β-actin-R	CCAGAGGCGTACAGGGATAG
Mcl-1L-F	GGAGGAGGAGGACGAGTTGTA
Mcl-1L-R	TGATGTCCAGTTTCCGAAGCA
Mcl-1S-F	GAGACGGCCTTCCAAGGAT
Mcl-1S-R	AGGTTGCTAGGGTGCAACTCT
DPYD-F	GAACTTGCCAAGAAGTCTGAGG
DPYD-R	ATGCCATGTGGACATGATAAAT
U6-RT	AACGCTTCACGAATTTGCGT
U6-F	CTCGCTTCGGCAGCACA
U6-R	AACGCTTCACGAATTTGCGT
si-Mcl-1-F	GGACUUUUAGAUUUAGUGAUU
si-Mcl-1-R	UCACUAAAUCUAAAAGUCCUU
si-DPYD-F	UAUUGUAACUGCACAUAAUGCUAGCUU
si-DPYD-R	GCUAGCAUUAUGUGCAGUUACAAUAUU

### 3.5. Cell Viability Assay

SMMC-7721/HepG2 cells were seeded into 96-well plates at a density of 4 × 10^3^ cells/well for 24 h before transfection. Cellular viability was measured at 24, 48, and 72 h post-transfection using 3-(4,5-dimethyl-2-thiazolyl)-2,5-diphenyl-2H-tetrazolium bromide (MTT) according to the manufacturer’s protocol. The absorbance at a wavelength of 490 nm was determined by a FLUOstar OPTIMA (BMG, Offenburg, Germany), which is positively related to the capacity of cellular viability. Each experiment was repeated three times.

### 3.6. Luciferase Assay

*PmirGLO*, *PmirGLO-Mcl-1-3′UTR-wt*, *pmirGLO-Mcl-1-3′UTR-mut*, *PmirGLO-DPYD-3′UTR-wt* and *PmirGLO-DPYD-3′UTR-mut* vectors were respectively co-transfected with miR-302b vector into 293 cells in a 96-well plate using lipofectamine 2000 (Invitrogen). Luciferase reporter gene assay was performed at 24 h after transfection using the Dual-Luciferase Reporter Assay System (Promega, Madison, WI, USA) according to the manufacturer’s protocol.

### 3.7. Cell Cycle Assays by Flow Cytometry

The HepG2 /SMMC-7721 cells were transfected with miR-302b and miR-ctrl vector in 12-well plates. Two days later, the cells were collected and fixed in 70% ethanol for 12 h at 4 °C, then washed by PBS, re-suspended in 200 μL of PBS containing 0.5 mg/mL RNase, 0.05% Triton X-100 and 10 μg/mL propidium iodide, incubated for 30 min at room temperature in the dark, and analyzed using a Flow Cytometer (FACSort; Becton Dickinson, San José, CA, USA).

### 3.8. 5-FU Sensitivity Assay

HepG2 or SMMC-7721 cells were seeded in triplicate onto 96-well plates and cultured for 24 h (4.0 × 10^3^ cells/well). They were then transfected with miR-ctrl plasmid, miR-302b plasmid, *Mcl-1* or *DPYD* siRNA and treated in 5-FU at a gradient concentration of 0, 0.2, 0.4, 0.8, 1.6, 3.2 μM and the other gradient concentration of 0, 0.2.5, 5, 10, 20, 40 μM. Cell proliferative activity was assessed, at 24, 48, and 72 h after 5-FU treatment, using MTT on FLUOstar OPTIMA (BMG). It was triplicate for each assay. The sequence of miR-302b plasmid has been shown above, and siRNA sequences are shown in Table 1.

### 3.9. Colony Assay

About 60 transfected HepG2/SMMC-7721 cells were seeded per well onto 6-well plates. When the cells had adhered to the bottom for 48 h, the culture medium in some wells for 5-FU sensitivity assay was replaced by the medium with the final concentration of 3.2 μM 5-FU. The cells were incubated for two weeks, and then were stained with 0.5% crystal violet for 30 min. Finally, the images were digitally captured.

### 3.10. Cell Apoptosis Assay by Flow Cytometry

Annexin V-FITC kit was used to determine the percentage of cells at the apoptotic phase. The transfected SMMC-7721 cells were incubated in the culture medium with 5-FU for 48 h at 37 °C. After being washed twice with PBS of around 4 °C, the cells were resuspended in binding buffer to the concentration of 1 × 10^6^ cells/mL. Then 100 μL of the cell suspension, 5 μL of Annexin V-FITC and 10 μL of Propidium Iodide (PI) were mixed into a tube of 5 mL, the cells were incubated in the dark for 15 min at room temperature. Then the cells were analyzed quickly by flow cytometry.

### 3.11. Western Blotting

Proteins were extracted using RIPA buffer, supplemented with protease inhibitor (invitrogen). Cell protein lysates were subjected to sodium dodecyl sulfate-polyacryl-amide gel electrophoresis (SDS-PAGE) and target proteins were detected with primary antibodies as follow: anti Mcl-1, anti Caspase-3 (1:1000; Cell Signaling Technology, Beverly, MA, USA), anti DPYD, anti Cleaved PARP (1:1000; Abcam, Cambridge, MA, USA), anti β-actin (1:4000; GeneTex, San Antonio, TX, USA) respectively. After incubation with corresponding horseradish peroxidase-conjugated secondary antibodies, and protein expression was normalized to the endogenous reference protein β-actin.

### 3.12. Statistical Analysis

Statistical analysis was performed by SPSS 16.0 (IBM, Armonk, NY, USA). Means for two groups were compared with an unpaired Student’s *t*-test (two-tailed). Comparisons of means for multiple groups against the miR-ctrl transfected HCC cell line group were analyzed with Dunnett’s multiple comparison tests. When the *p* value was less than 0.05, differences were considered significant.

## 4. Conclusions

In conclusion, we have shown that miR-302b can enhance 5-FU chemotherapy sensitivity in liver cancer cells. This biological function might be attributed to targeting *Mcl-1* and *DPYD*. Because of its low level of expression in HCC cells, miR-302b will be a potential candidate molecular to improve the efficiency of chemotherapy in the treatment of advanced HCC patients after development of better gene delivery systems.

## Supplementary Materials

Supplementary materials can be accessed at: http://www.mdpi.com/1422-0067/16/10/23668/s1.
