# Supplementary material for: MicroRNA-302b Enhances the Sensitivity of Hepatocellular Carcinoma Cell Lines to 5-FU via Targeting Mcl-1 and DPYD
Source: Int J Mol Sci. 2015 Oct 6;16(10):23668–82. doi: 10.3390/ijms161023668 (PMC4632720; doi:10.3390/ijms161023668)
Supplement: Supplementary file 1 [file ijms-16-23668-s001.pdf]

Supplementary Information

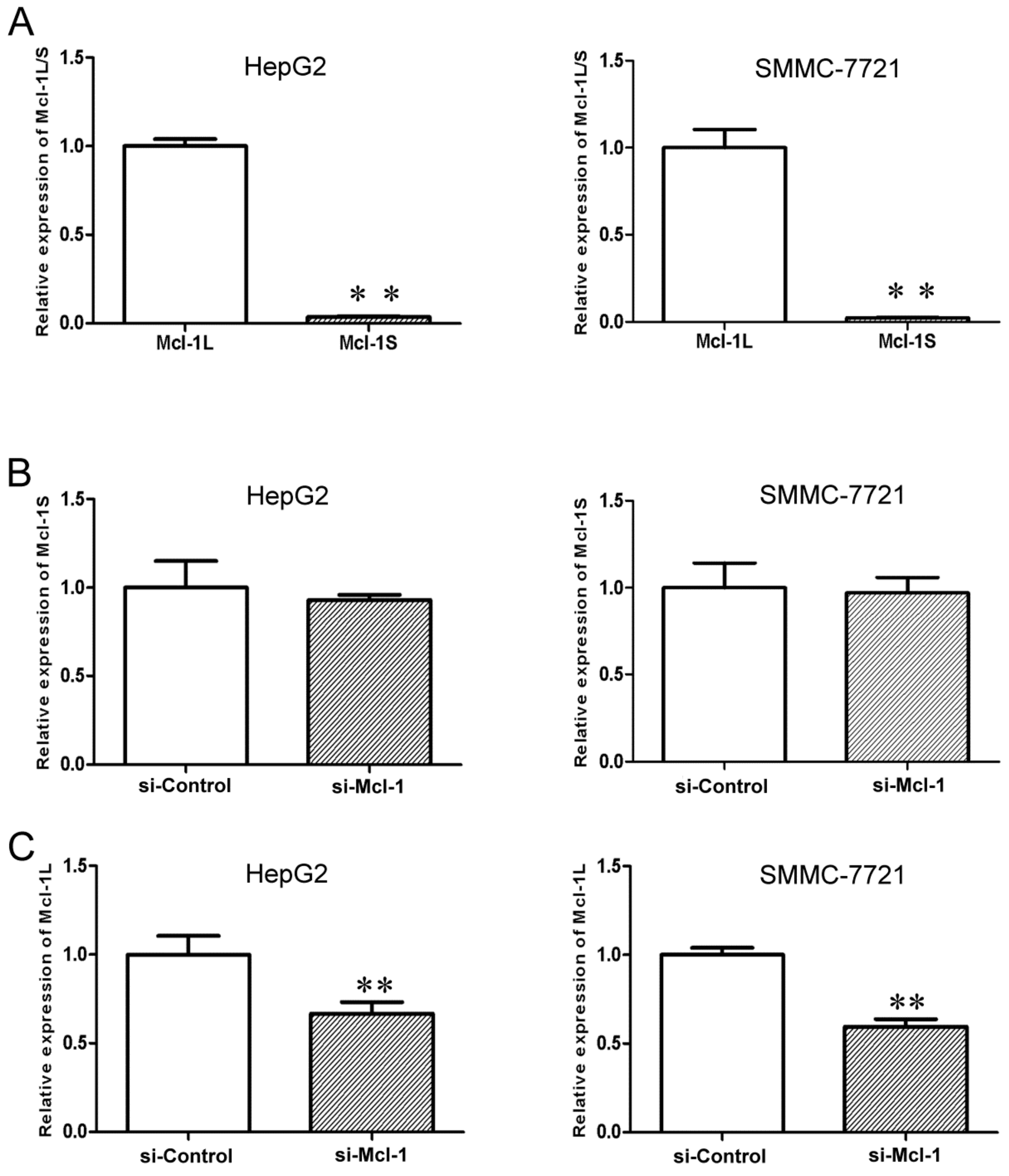

Figure S1. *Mcl-1S* little influence the inhibiting effect of *Mcl-1* siRNA. (**A**) QRT-PCR showed that a huge difference in endogenous expression level between *Mcl-1L* and *Mcl-1S* in HepG2/SMMC-7721 cell lines; (**B**) *Mcl-1S* decreased little after transfection of *Mcl-1* siRNA into HepG2/SMMC-7721; (**C**) *Mcl-1L* decreased obviously after transfection of *Mcl-1* siRNA into HepG2/SMMC-7721. Error bars represent the S.D; ** $p < 0.01$, *vs.* the corresponding controls.
